# Amelioration of Non-Alcoholic Steatohepatitis by *Atractylodes macrocephala* Polysaccharide, Chlorogenic Acid, and Geniposide Combination Is Associated With Reducing Endotoxin Gut Leakage

**DOI:** 10.3389/fcimb.2022.827516

**Published:** 2022-07-05

**Authors:** Jing Leng, Hua-jie Tian, Yi Fang, Yi-yang Hu, Jing-hua Peng

**Affiliations:** ^1^ Institute of Liver Diseases, Shuguang Hospital affiliated to Shanghai University of Traditional Chinese Medicine, Shanghai, China; ^2^ Key Laboratory of Liver and Kidney Diseases (Shanghai University of Traditional Chinese Medicine), Ministry of Education, Shanghai, China; ^3^ Shanghai Key Laboratory of Traditional Chinese Clinical Medicine, Shanghai, China; ^4^ School of Basic Medical Sciences, Shanghai University of Traditional Chinese Medicine, Shanghai, China; ^5^ Institute of Clinical Pharmacology, Shuguang Hospital Affiliated to Shanghai University of Traditional Chinese Medicine, Shanghai, China

**Keywords:** *Atractylodes macrocephala* polysaccharide, chlorogenic acid, geniposide, non-alcoholic steatohepatitis, lipopolysaccharide, intestinal tight junctions

## Abstract

Gut-derived lipopolysaccharide (LPS) leaking through the dysfunctional intestinal barrier contributes to the onset of non-alcoholic steatohepatitis (NASH) by triggering inflammation in the liver. In the present study, a combination consisting of *Atractylodes macrocephala* polysaccharide (A), chlorogenic acid (C), and geniposide (G) (together, ACG), was shown to ameliorate NASH in mice and reduce hepatic LPS signaling and endotoxemia without decreasing the abundance of identified Gram-negative bacteria through restoring the intestinal tight junctions. Our data indicated that inhibition of LPS gut leakage by the ACG combination contributed to its amelioration of NASH.

## 1 Introduction

Non-alcoholic fatty liver disease (NAFLD) is the subject of increasing concern not only in clinical practice but also in the basic research field because of its high global prevalence and close association with metabolic comorbidities including obesity, diabetes mellitus, and cardiovascular disease. In the wide spectrum of NAFLD histological features, non-alcoholic steatohepatitis (NASH) is an important pathological stage bridging steatosis to fibrosis (G.B.D. Mortality, 2016). Many drugs for NASH are undergoing clinical trials, including peroxisome proliferator-activated receptor agonists, mitochondrial pyruvate carrier inhibitor, ketohexokinase inhibitor, farnesoid X receptor agonist, liver X receptor alpha inhibitor, fibroblast growth factor analogs, glucagon-like peptide and glucose-dependent insulinotropic peptide receptor analogs or agonists, thyroid hormone receptor–selective agonist, fatty acid synthesis enzyme inhibitors, glucocorticoid receptor antagonist, and growth hormone releasing hormone analog (Negi et al., 2022). However, so far, only pioglitazone and vitamin E were recommended to treat biopsy-proven NASH (Chalasani et al., 2018), due to the undisclosed endpoints of these clinical trials. Meanwhile, the side effects also limited the use of these drugs.

In China, traditional Chinese medicine is considered as an important alternative strategy for the treatment of NASH. The Branch of Gastrointestinal Diseases, China Association of Chinese Medicine, has published an expert consensus on traditional Chinese medicine diagnosis and treatment of NAFLD and recommended traditional Chinese medicinal herbs and treatment principles for NAFLD ((Branch of Gastrointestinal Diseases China Association of Chinese Medicine, 2017). We previously screened many active components of traditional Chinese herbs on NAFLD in mice induced by a high-fat diet (HFD) and obtained a combination consisting of *Atractylodes macrocephala* polysaccharide (A, 266.67 mg/kg body weight), chlorogenic acid (C, 3.3 mg/kg body weight), and geniposide (G, 45 mg/kg body weight) (Meng et al., 2016). *Atractylodes macrocephala* polysaccharide is the primary component extracted from *Atractylodis Macrocephalae* Rhizoma, which was traditionally used to improve the function of the digestive system and is recorded in “Compendium of Materia Medica” (Bencao Gangmu), a pharmaceutical monograph written in the Qing dynasty of ancient China. Chlorogenic acid and geniposide are derived from Yin-Chen-Hao Tang, which was traditionally used to reduce heat and dampness and has been demonstrated to protect hepatocytes (Dong et al., 2012). ACG combination was found to ameliorate hepatic lipid accumulation and inflammatory infiltration significantly (Meng et al., 2016).

As the role of gut microbiota and intestinal barrier dysfunction in the onset of NASH are increasingly recognized, the gut-derived lipopolysaccharide (LPS) has been demonstrated to participate in the pathogenesis of NASH (Dai and Wang, 2015). In the present study, the effect of the ACG combination on NASH was evaluated and its potential acting mechanisms on hepatic inflammation caused by gut-derived LPS was investigated. Sodium butyrate (NaB) was used as the positive control drug, since it has been shown to protect intestinal tight junctions (Wang et al., 2012) and improve steatohepatitis (Zhou et al., 2017).

## 2 Materials and Methods

### 2.1 Material

*Atractylodes macrocephala* polysaccharide, chlorogenic acid {International Union of Pure and Applied Chemistry (IUPAC) name: (1S,3R,4R,5R)-3-[(E)-3-(3,4-dihydroxyphenyl)prop-2-enoyl]oxy-1,4,5-trihydroxycyclohexane-1-carboxylic acid; purity, >98%}, and geniposide {IUPAC name: methyl (1S,4aS,7aS)-7-(hydroxymethyl)-1-[(2S,3R,4S,5S,6R)-3,4,5-trihydroxy-6-(hydroxymethyl)oxan-2-yl]oxy-1,4a,5,7a-tetrahydrocyclopenta[c]pyran-4-carboxylate; purity, >98%} were purchased from Shanghai Winherb Medical Technology Co., Ltd. (Shanghai, China). The botanical names have been updated according to The Plant List database (http://www.theplantlist.org).

### 2.2 Preliminary Chemical Analysis of *Atractylodes macrocephala* Polysaccharide

As described previously (Saha and Brewer, 1994), *Atractylodes macrocephala* polysaccharide was analyzed to contain 73.64% total carbohydrate (**Supplementary Table 1**, **Supplementary Figure 1**). Gas chromatograph–mass spectrometer (Thermo Fisher Scientific, Inc., FL, USA) was employed to analyze the carbohydrate composition of *Atractylodes macrocephala* polysaccharide in accordance with the protocol, which showed 5.76% arabinose and 94.24% glucose (**Supplementary Figure 2**).

### 2.3 Study Setting

Male C57BL/6 mice (6 weeks old; Shanghai Experimental Animal Center of Chinese Academy of Sciences, Shanghai, China) were randomly divided into control (n = 9, control diet, D12450B, 10% kcal from fat; Research Diets, Inc., NJ, USA), HFD (n = 9, D12492, 60% kcal from fat; Research Diets, Inc., NJ, USA), ACG (n = 9, fed with HFD), and NaB (n = 9, fed with HFD) groups. At the beginning of the 13th week, mice in ACG and NaB groups were, respectively, administrated with the ACG combination (*Atractylodes macrocephala* polysaccharide, 266.67 mg/kg body weight; chlorogenic acid, 3.3 mg/kg body weight; and geniposide, 45 mg/kg body weight, daily) (Meng et al., 2016) and NaB (200 mg/kg body weight daily; Sigma-Aldrich, USA) (Zhou et al., 2017) intragastrically for 4 weeks. The others were administrated with double distilled water. At the end of the 16th week, the blood from the caudal vena cava, liver, and colon tissue and the colonic feces were harvested for assay. All animals received humane care and the animal study protocols were approved by the animal studies ethics committee of the Shanghai University of Traditional Chinese Medicine.

### 2.4 Histopathology Examination

The histological changes were illustrated *via* hematoxylin and eosin staining (Nanjing Jiancheng Institute of Bio Engineering, Inc., Nanjing, China). The NAFLD activity score (NAS) system was employed to evaluate hepatic histology, and NAS of > 5 is diagnosed as NASH (**Supplementary Table 2**) (Kleiner et al., 2005). The colonic histological injury was evaluated by the parameters including epithelial cell injury/loss, mucin (goblet cell) loss, mucosal edema, and the degree of inflammatory cells within the lamina propria and in the epithelial layer (intraepithelial lymphocytes) (Keshavarzian et al., 2001). The hepatic collagen was visualized by Sirius Red staining. The lipid droplets in hepatocyte were visualized *via* Oil Red staining (Sigma, MO, USA) on frozen tissue.

### 2.5 Alanine Aminotransferase and Triglyceride Assays

The assay kits of alanine aminotransferase (ALT) (Nanjing Jiancheng Bioengineering Institute, Nanjing, China) and triglyceride (TG) (Dongou Diagnostic Products Co., Ltd., Zhejiang, China) were used to determine ALT activity in the plasma and hepatic TG content according to the instructions.

### 2.6 Real-Time Polymerase Chain Reaction

Total RNA was extracted from the liver tissue (Total RNA Extractor, Sangon Biotech, Inc., Shanghai, China) and then reversely transcribed into complementary DNA (cDNA) (iScript™ cDNA Synthesis Kits, Bio-Rad, CA, USA). With the special primers (**Supplementary Table 3**) and commercial kit (TB Green™ Premix Ex Taq™, TaKaRa Bio, Inc., Japan), real-time polymerase chain reaction (PCR) was conducted to detect mRNA expression of hepatic collagen I, IV, CD14, and myeloid differentiation primary response 88 (MyD88) (Applied Biosystems ViiA7) (Thermo Fisher Scientific, CA, USA). The expression of target gene was calculated by the delta-delta Ct method and presented as the fold changes relative to control.

### 2.7 Assay for LPS and D-Lactic Acid Content

The blood samples collected from the caudal vena cava in pyrogen-free and heparin-pretreated tubes were centrifuged (500*g*, 15 min, 4°C) to obtain plasma. LPS content was measured by a Pyrochrome Limulus Amebocyte Lysate kit (Associates of Cape Cod, East Falmouth, MA). The PicoProbe D-Lactate Assay Kit (Fluorometric) (ab174096, Abcam, MA, USA) was employed to measure D-lactic acid in the plasma.

### 2.8 Immunofluorescence Staining

The immunofluorescence staining of hepatic F4/80, colonic ZO-1, and Occludin was conducted as described previously (Peng et al., 2018). The primary and secondary antibodies were listed in **Supplementary Table 4**. The fluorescence signal of target protein was visualized and observed under laser scanning confocal microscope (OLYMPUS-FV10i, Olympus Corporation, Tokyo, Japan).

### 2.9 Enzyme-Linked Immunosorbent Assay

Commercial enzyme-linked immunosorbent assay (ELISA) kits were used for assay of LPS-binding protein (LBP) (CKM043, Cell Sciences, Inc., MA, USA), tumor necrosis factor (TNF)–α (MBS49535, MyBioSource, Inc., CA, USA), and interleukin (IL)–1β (MBS036031, MyBioSource, Inc., CA, USA) as described previously (Peng et al., 2018).

### 2.10 Gut Microbiota Analysis

The composition of gut microbiota was analyzed by sequencing of bacterial 16S rRNA (V3–V4 region) in colonic feces, which was a contract service offered by Shanghai OE Biotech. Co., Ltd. (Shanghai, China). The total genomic DNA of gut microbiota was extracted and purified according to the previous protocol (Leng et al., 2020). The PCR amplification of V3–V4 variable regions of 16S rRNA genes was conducted with universal primers (343F: 5′-TACGGRAGGCAGCAG-3′; 798R: 5′-AGGGTATCTAATCCT-3′). Please find the dataset at https://data.mendeley.com/datasets/fzfnyxn5rm/1. As described previously (Leng et al., 2020), the same operational taxonomic units (OTUs) were consisted of valid tags with 97% similarity *via* Vsearch software (Edgar et al., 2011). OTU was annotated and blasted against Silva database version 123 (Quast et al., 2013) by using Ribosomal Database Project (RDP) classifier (confidence threshold was 70%).

Quantitative Insights Into Microbial Ecology (QIIME) software package was employed to analyze alpha diversity including Chao, Shannon–Wiener, Simpson, and Good’s coverage index. Beta-diversity was measured *via* Bray–Curtis distance matrix and then build principal coordinates analysis (PCoA) plots with QIIME (1.8.0). The distance among the samples was calculated with the unweighted pair group method with arithmetic mean (UPGMA) clustering method. Analysis of similarities (ANOSIM) was performed to identify the difference of microbiota composition among the groups.

### 2.11 Western Blot Analysis

The protein expression of tight junctions including ZO-1, Occludin, and Claudin-1 in the colon tissue was detected *via* Western blot analysis as described previously (Peng et al., 2009). The primary and fluorescence-tagged secondary antibodies were summarized in **Supplementary Table 4**. The fluorescent signal of target protein was obtained *via* scanning the membranes by Odyssey quantitative Western blot near-infrared system (LI-COR Biosciences, NE, USA), and the intensity of target band was calculated by using Odyssey application software version 3.0 (LI-COR Biosciences, NE, USA) and corrected with the intensity of β-actin. Data were represented as the fold changes relative to control.

### 2.12 Statistical Analysis

After analysis of variance test, the Student’s t-test was employed to analyze the statistical significance between two groups. Data were expressed as mean ± standard deviation. The Kruskal–Wallis H-test was employed to analyze the statistic difference among more than two groups for the non-parametric data. Significance was accepted at the level of P < 0.05. The data of 16S sequencing were analyzed by bioinformatics methods mentioned in Section 2.10.

## 3 Results

### 3.1 ACG Combination Alleviated NASH Induced by HFD

**Figure 1:**
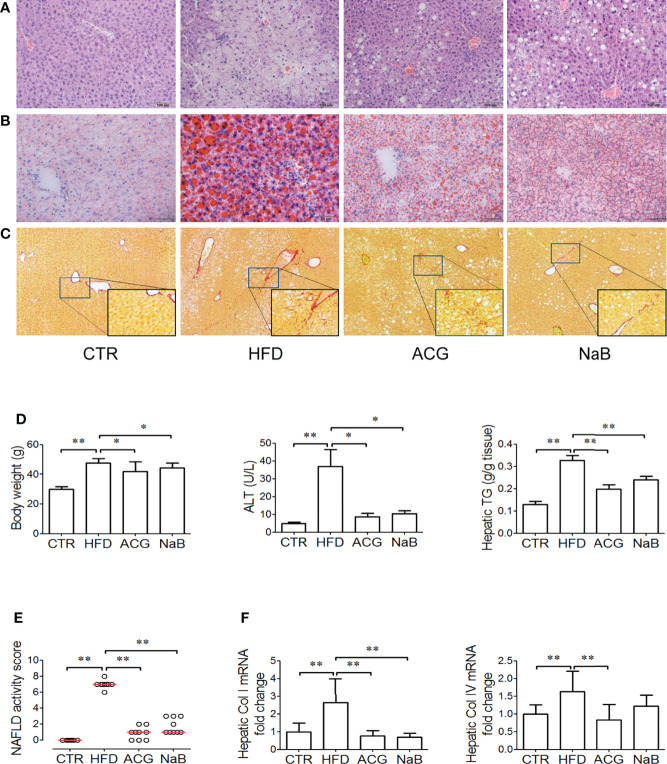
Effects of *Atractylodes macrocephala* polysaccharide, chlorogenic acid, and geniposide combination on non-alcoholic steatohepatitis induced by high-fat diet. **(A)** Hematoxylin and eosin staining of liver tissue (×200 magnification). **(B)** Oil Red staining of liver tissue (for visualization of lipid droplets, ×200 magnification). **(C)** Sirius Red staining of liver tissue (for visualization of collagen, ×100 magnification). **(D)** Body weight, ALT activity in the plasma, and hepatic TG content. **(E)** NAFLD activity score. Data represented the median of groups and individual NAS of samples. **(F)** Hepatic mRNA expression of collagen I and collagen IV. CTR, control, HFD, high-fat diet; ACG, combination consisting of *Atractylodes macrocephala* polysaccharide, chlorogenic acid, and geniposide; NaB, sodium butyrate; NAFLD, non-alcoholic fatty liver disease. *p < 0.05 and **p < 0.01.

Obvious steatosis, ballooning, and inflammation were observed in mice in the HFD group (**Figures 1A, B**). The median of NAS increased up to 7 in the HFD group (p < 0.01, vs. control), which indicated NASH had been established (**Figure 1E**). Consistently, body weight, serum ALT, and hepatic TG all increased (p < 0.01, vs. control) in the HFD group (**Figures 1D, E**). With treatment with ACG or NaB, the pathological changes in the liver including steatosis, ballooning, and inflammation were ameliorated (**Figures 1A, B**). The median of NAS in the ACG and NaB group decreased to 1, which indicated that NASH induced by HFD was alleviated by ACG or NaB treatment (p < 0.01, vs. HFD) (**Figure 1E**). Simultaneously, body weight (p < 0.05, vs. HFD), serum ALT (p < 0.05, vs. HFD), and hepatic TG (p < 0.01, vs. HFD) all decreased with the administration of ACG or NaB compared with those in the HFD group (**Figures 1D, E**).

With HFD feeding for 16 weeks, mild fibrosis was observed in the perisinus area (**Figure 1C**), which was consistent with the increased mRNA expression of collagen I and IV (p < 0.01, vs. control) in the HFD group (**Figure 1F**). In Sirius Red–stained sections, the difference of fibrosis was not obvious between the ACG- or NaB-treated group and the HFD group (**Figure 1C**), but the mRNA expression of collagen I and IV both decreased (p < 0.01, vs. HFD) with ACG or NaB administration (**Figure 1F**).

### 3.2 ACG Combination Ameliorated LPS Signaling in the Liver and Endotoxemia Induced by HFD

**Figure 2:**
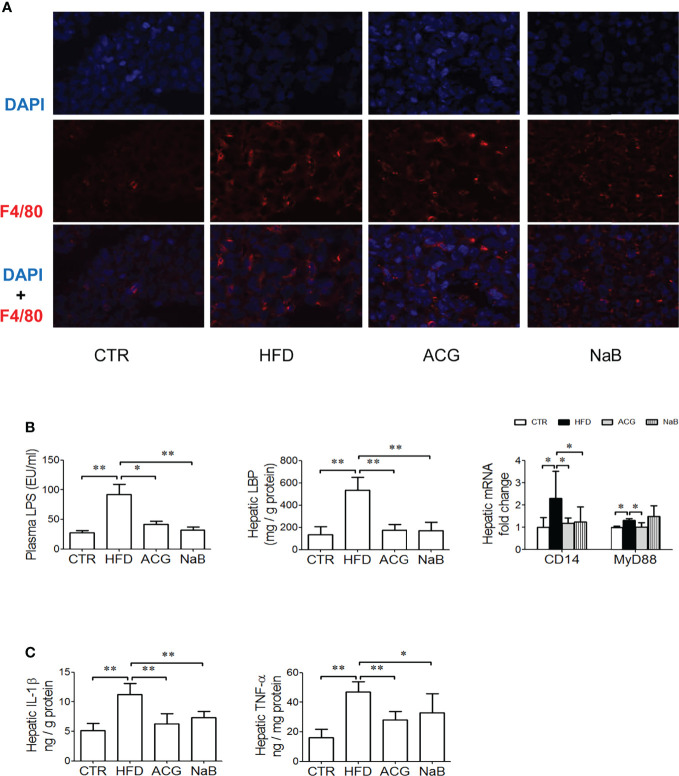
Effects of *Atractylodes macrocephala* polysaccharide, chlorogenic acid, and geniposide combination on lipopolysaccharide signaling and endotoxemia in non-alcoholic steatohepatitis induced by high-fat diet. **(A)** Immunofluorescence staining for F4/80 in the liver tissue (×600 magnification). **(B)** The LPS level in the plasma, the hepatic LBP content, and the mRNA expression of CD14 and MyD88 in the liver tissue. **(C)** The hepatic content of IL-1β and TNF-α. CTR, control; HFD, high-fat diet; ACG, combination consisting of *Atractylodes macrocephala* polysaccharide, chlorogenic acid, and geniposide; NaB, sodium butyrate; LPS, lipopolysaccharide; LBP, lipopolysaccharide-binding protein; IL-1β, interleukin-1β; TNF-α, tumor necrosis factor–α. *p < 0.05 and **p < 0.01.

In the HFD group, the LPS level in the plasma increased (p < 0.01, vs. control) accompanied with more Kupffer cells infiltration in the liver tissue visualized by the stronger positive- staining of F4/80 (**Figures 2A, B**). As expected, the hepatic LBP content (p < 0.01, vs. control), the mRNA expression of CD14 and MyD88 (p < 0.05, vs. control), as well as the hepatic IL-1β and TNF-α content (p < 0.01, vs. control) (**Figure 2C**) increased in the HFD group compared with that in the control.

With the treatment with ACG or NaB, endotoxemia ameliorated (p < 0.05, ACG vs. HFD, p < 0.01, NaB vs. HFD), and rarer positive staining of F4/80 was observed in the liver. At the same time, hepatic LBP (p < 0.01, vs. HFD), mRNA expression of CD14 and MyD88 (p < 0.05, vs. HFD), and hepatic TNF-α (p < 0.01, vs. HFD) and IL-1β (p < 0.01, ACG vs. HFD, p < 0.05, NaB vs. HFD) were all lower than in the HFD group. (**Figures 2B, C**).

### 3.3 ACG Combination Changed the Community Structure of Gut Microbiota Without Decreasing Relative Abundance of Gram-Negative Bacteria in NASH

To determine whether ACG treatment reduced the abundance of Gram-negative bacteria, the source of LPS, in the intestine, the community structure of gut microbiota was assayed. The alpha diversity of gut microbiota illustrated by Chao 1, observed species, Simpson, and Shannon index indicated less species diversity in samples of the ACG group than in the HFD and control groups (**Supplementary Table 5**). The sequencing depth covered rare new phylotypes and most of the diversity, which was supported by Good’s coverage estimator and Specaccum species accumulation curve (**Supplementary Figure 3**).

**Figure 3:**
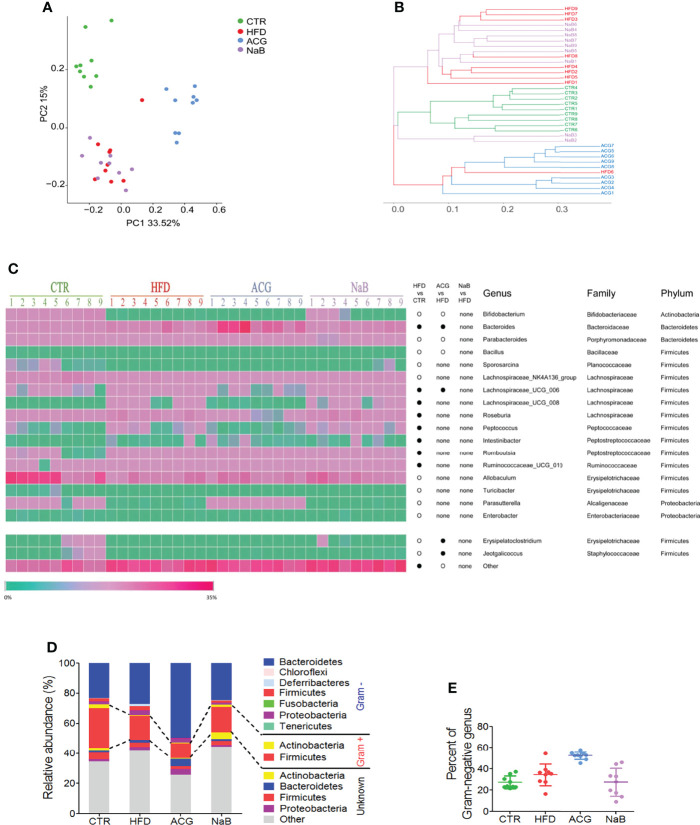
Effects of *Atractylodes macrocephala* polysaccharide, chlorogenic acid, and geniposide combination on the community construction of gut microbiota in non-alcoholic steatohepatitis induced by high-fat diet. The diversity of gut microbiota was analyzed by sequencing of bacterial 16S rRNA (V3–V4 region). **(A)** Bray–Curtis–based principal coordinates analysis (PCoA) analysis of the gut microbiota composition. **(B)** The unweighted pair group method with arithmetic mean (UPGMA) clustering of the gut microbiota. **(C)** The relative abundance of genus in the gut microbiota. •, more abundant; ○, less abundant. **(D)** The average phylum distribution of the gut microbiota. The information of Gram staining of bacteria was identified on the NIBC database of taxonomy (https://www.ncbi.nlm.nih.gov/taxonomy/). **(E)** Relative abundance of Gram-negative bacteria in gut microbiota. CTR, control; HFD, high-fat diet; ACG, combination consisting of *Atractylodes macrocephala* polysaccharide, chlorogenic acid, and geniposide; NaB, sodium butyrate.

With Bray–Curtis–based PCoA, three distinct clusters of microbiota composition were observed (**Figure 3A**). It was intuitively visualized by the UPGMA clustering analysis that most samples of the HFD and NaB groups were clustered together and far from that of the control and ACG group (**Figure 3B**). Although ACG samples were clustered with one HFD sample, a significant difference between the HFD and ACG groups was detected (R statistic = 0.75; ANOSIM, 999 permutations; P = 0.001).

Compared with the control group, the relative abundance of 19 identified genera was statistically changed by HFD feeding (p < 0.05), including one genus in phylum of Actinobacteria, two in Bacteroidetes and Proteobacteria, respectively, and 14 genera in Firmicutes (**Figure 3C**). With the treatment with the ACG combination, the relative abundance of the seven identified genera changed compared with that of the HFD group (p < 0.05), in which the decreased relative abundance of *Erysipelatoclostridium* and *Jeotgalicoccus* in the HFD group was restored in the ACG group (**Figure 3C**). However, with the NaB treatment, no obvious difference in the relative abundance of genera was observed compared with that of the HFD group (p > 0.05) (**Figure 3C**).

To investigate whether regulation of the composition of gut microbiota by the ACG combination contributed to its inhibition of gut leakage of LPS, the relative abundance of the Gram-negative bacteria was analyzed. In the HFD group, the relative abundance of identified Gram-negative genera was increased but not statistically significant compared with that in the control group. However, in the ACG group, the relative abundance of identified Gram-negative genera did not decrease significantly compared with that in the HFD group, which indicated that other mechanisms participated in the amelioration of endotoxemia. (**Figures 3D, E**; **Supplementary Table 6**).

### 3.4 ACG Combination Restored the Protein Expression of Intestinal Tight Junction in NASH

**Figure 4:**
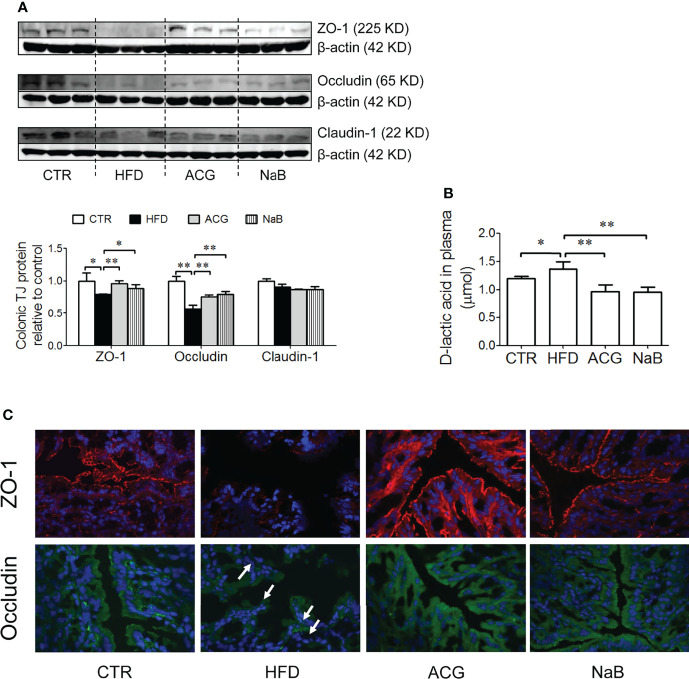
Effects of *Atractylodes macrocephala* polysaccharide, chlorogenic acid, and geniposide combination on tight junction protein expression in the colon tissue and D-lactic acid in the plasma in non-alcoholic steatohepatitis induced by high-fat diet. **(A)** Protein expression of ZO-1, Occludin, and Claudin-1 in the colon tissue detected by Western blot and the intensity analysis of target band. The expression of target proteins were corrected by β-actin and represented as the fold changes relative to control. **(B)** Immunofluorescence staining of ZO-1 and Occludin in the colon (×600 magnification). White arrow points out the tight junction disruption. **(C)** D-lactic acid content in the plasma. CTR, control; HFD, high-fat diet; ACG, combination consisting of *Atractylodes macrocephala* polysaccharide, chlorogenic acid, and geniposide; NaB, sodium butyrate. *p < 0.05 and **p < 0.01.

With HFD feeding, few pathological changes were found in the colon under the light microscope, but the protein expression of tight junctions including ZO-1 and Occludin was obviously downregulated (p < 0.05) (**Figures 4A, C**). The protein expression of Claudin-1 in the colon tissue was not different among groups (**Figures 4A, C**). With the treatment of the ACG combination (p < 0.01, vs. HFD) or NaB (p < 0.05, vs. HFD), ZO-1 and Occludin protein in the colon tissue was restored, which indicated that the inhibition on LPS gut leakage by the ACG combination or NaB was probably associated with the protection on the intestinal barrier function (**Figures 4A, C**). Consistently, the marker of gut leakage, D-lactic acid in the plasma increased in the HFD group and was inhibited by the ACG or NaB treatment (**Figure 4B**).

## 4 Discussion

The causal role of intestinal microbiota in NAFLD has been demonstrated. Transplantation of the gut microbiota of NAFLD mice to the germ-free mice caused steatosis in the liver tissue (Le Roy et al., 2013). A mixture of *Streptococcus thermophilus* and several species of *Lactobacillus* and *Bifidobacterium* improved the liver histology and serum ALT and reduce the hepatic content of fatty acid in ob/ob mice (Li et al., 2003).

In our study, with HFD feeding, the community structure of intestinal microbiota was obviously changed and accompanied by NASH. Compared with the control group, the relative abundance of genera *Bacteroides*, *Peptococcus*, *Intestinibacter*, and *Romboutsia* and members of family Lachnospiraceae (*Lachnospiraceae_UCG_006*, *Lachnospiraceae_UCG_008*, and *Roseburia*) and Ruminococcaceae (*Ruminococcaceae_UCG_010*) were upregulated with HFD feeding. The functions of these bacteria have been reported previously except genera *Intestinibacter*. Genus *Bacteroides*, Gram-negative, produces branched-chain amino acid (Canfora et al., 2019), which is correlated to obesity and insulin resistance in animal and human models (Lynch and Adams, 2014; Moran-Ramos et al., 2017). *Bacteroides* express gadB/gadC gene coding Gamma-amino butyric acid (GABA) (Yunes et al., 2016), which was reported to be higher in the brain of patients of type 2 diabetes (Farzi et al., 2019). Genus *Peptococcus* ferments amino acids to produce polyamines (Canfora et al., 2019), which improve glucose homeostasis and insulin sensitivity and ameliorate obesity in mouse models (Ramos-Molina et al., 2019). However, mice in the HFD group were still insulin-resistant (**Supplementary Figure 4A**) with much higher fasting glucose and insulin levels compared with those in the control group, which indicated that this relatively abundant genus *Peptococcus* (the relative abundance was around 0.5%) in the HFD group was not sufficient to defend against the phenotype induced by HFD. Genus *Romboutsia* is beneficial and contains fermentative bacteria (Hasan et al., 2018). The Lachnospiraceae and Ruminococcaceae family contains protective gut commensal strains (Sanders et al., 2019), producing short-chain fatty acids (SCFAs) (Canfora et al., 2019) to provide energy for enterocytes locally and maintain theintegrity of the intestinal epithelial barrier (Kaiko et al., 2016), whereas the Lachnospiraceae family also contains a strain that significantly increases fasting blood glucose in colonized germ-free *ob/ob* mice (Kameyama and Itoh, 2014).

On the other hand, compared with the control group, the relative abundance of genera *Allobaculum*, *Turicibacter*, *Parasutterella*, *Enterobacter*, *Sporosarcina*, *Bacillus*, *Parabacteroides*, *Bifidobacterium*, *Erysipelatoclostridium*, and *Jeotgalicoccus* and members of the Lachnospiraceae family (*Lachnospiraceae_NK4A136_group*) were downregulated by HFD feeding. Genus *Allobaculum* (Zhang et al., 2015) and *Turicibacter* (Bosshard et al., 2002) and members of the Lachnospiraceae family (Bajaj, 2019) produce SCFAs (Bosshard et al., 2002; Teixeira et al., 2018). Genera *Sporosarcina* (Priyodip and Balaji, 2019) and *Bacillus* (Bajaj, 2019) are potential probiotics. *Bifidobacterium* is probiotics (Stadlbauer et al., 2008), which is associated with amelioration of NASH (Malaguarnera et al., 2012). *Parabacteroides* (Yunes et al., 2016) and *Bifidobacterium* (Yunes et al., 2016) express GABA. Consistently, *Parasutterella* decreased with HFD feeding (Kreutzer et al., 2017) and increased with sugar (Noble et al., 2017) and alcohol consumption (Zhang et al., 2017). *Enterobacter* is Gram-negative and contains endotoxin (Bajaj, 2019). Genus *Erysipelatoclostridium* contains opportunistic strains (Shao et al., 2017).

After treatment with the ACG combination, the composition of gut microbiota was significantly different from the HFD group, whereas NaB had no obvious effects on the composition of gut microbiota. NaB was reported to regulate gut microbiota in NAFLD and inflammatory bowel disease, but it was administrated with 200–300 mg/kg/day for 8–12 weeks in those experiments (Zhou et al., 2017; Fang et al., 2019; Yu et al., 2019; Facchin et al., 2020) In the ACG-treated group, the relative abundance of the genus *Bacteroides* and members of the Lachnospiraceae family (*Lachnospiraceae_UCG_006*) increased, whereas genera *Bifidobacterium*, *Parabacteroides*, and *Bacillus* decreased compared with the HFD group. The decreased abundance of genera *Erysipelatoclostridium* and *Jeotgalicoccus* by HFD feeding was restored by the ACG treatment. Meanwhile, the ACG treatment appeared to reduce the alpha diversity of intestinal microbiome, which is usually recognized as a negative index of intestinal health. However, in actual fact, the alpha diversity of intestinal microbiomes in NAFLD was reported ambiguously. Lower alpha diversity was observed in the gut microbiome of patients with NAFLD (Schwimmer et al., 2019; Pan et al., 2021). However, it was also reported that there was no significant difference in alpha diversity in the gut microbiotomes of patients with NAFLD and healthy controls (Zhao et al., 2019; Iwaki et al., 2021). In addition, in female patients with NAFLD, even higher microbial alpha diversity was observed (Shi et al., 2021).

It appears hard to explain the increased gut leakage of LPS by HFD feeding and the decreased gut leakage of LPS by the ACG treatment only based on the identified functions of the differential bacteria. Hence, we looked closely into the relative abundance of identified Gram-negative bacteria, the source of LPS. The abundance of Gram-negative bacteria in the HFD group increased with no statistical significance compared with that of the control group. However, as unexpected, the relative abundance of identified Gram-negative bacteria in the ACG combination group was not decreased compared to that in the HFD group, which cannot explain the endotoxemia caused by HFD feeding and the amelioration of endotoxemia by the ACG combination in NASH.

It has been demonstrated that compared with the healthy subjects, significantly increased intestinal permeability was observed in patients or experimental animals with NASH (Dai and Wang, 2015), which appears to be caused by the disruption of intercellular tight junctions in the intestine, the key factor of gut mucosa barrier function (Miele et al., 2009). In NASH, the disruption of tight junction allows bacteria and toxic molecules, such as LPS, to translocate from the intestine to the portal vein and eventually to the liver. Increased LPS entering the liver recruits and activates Kupffer cells to produce proinflammatory cytokines. As shown in the present study, the increased F4/80 positive area, the mRNA expression of CD14 and MyD88, and the levels of LBP, IL-1β, and TNF-α in the liver were accompanied with an increased LPS level in the plasma with HFD feeding, which indicated activation of Kupffer cells in the liver tissue in NASH.

In the present study, the disruption of tight junction in the colon was detected in the HFD-fed mice accompanied with increased NASH. The ACG combination restored the protein expression of tight junction in parallel with the inhibitory effect on LPS gut leakage, which indicated that the amelioration of endotoxemia by the ACG combination was associated with its protection on intestinal tight junction in NASH. NaB also restored the protein expression of intestinal tight junctions and ameliorated endotoxin signaling including downregulation on hepatic LBP, CD14, IL-1β, and TNF-α, although it had no effect on hepatic MyD88.

Consistently, an inhibitor of intestinal permeability was demonstrated to have a positive effect on patients with NAFLD in a recently finished phase II clinic trial (Kessoku et al., 2020). On the other hand, NaB protected the intestinal mucosal barrier in NASH but had no regulatory effect on the community structure of gut microbiota.

## Data Availability Statement

The datasets of bacterial 16S rRNA sequencing presented in this study can be found in online repositories. The names of the repository/repositories and accession number(s) can be found at https://data.mendeley.com/datasets/fzfnyxn5rm/1, DOI: 10.17632/fzfnyxn5rm.1.

## Ethics Statement

The animal study was reviewed and approved by animal studies ethics committee of the Shanghai University of Traditional Chinese Medicine.

## Author Contributions

JL conducted the animal experiments. HT conducted ELISA. YF performed real-time PCR. YH provided advices and comments during the project design and data integration. JP designed the project, analyzed and integrated data, and wrote the manuscript. All authors contributed to the article and approved the submitted version.

## Funding

This work was supported by National Natural Science Foundation of China (nos. 81673750, 81673765, 81370094, and 81001575), Science and Technology Commission Shanghai Municipality (17PJ1408900), Shuguang Hospital affiliated to Shanghai University of Traditional Chinese Medicine (SGXZ-201911).

## Conflict of Interest

The authors declare that the research was conducted in the absence of any commercial or financial relationships that could be construed as a potential conflict of interest.

## Publisher’s Note

All claims expressed in this article are solely those of the authors and do not necessarily represent those of their affiliated organizations, or those of the publisher, the editors and the reviewers. Any product that may be evaluated in this article, or claim that may be made by its manufacturer, is not guaranteed or endorsed by the publisher.

## Abbreviations

ACG formula, *Atractylodes macrocephala* polysaccharide, chlorogenic acid, and geniposide; ALT, alanine aminotransferase; cDNA, complementary DNA; ELISA, enzyme-linked immunosorbent assay; GABA, gamma-amino butyric acid; HFD, high-fat diet; LBP, lipopolysaccharide-binding protein; IL-1β, interleukin-1β; LPS, lipopolysaccharide; MyD88, myeloid differentiation primary response 88; NaB, sodium butyrate; NAFLD, non-alcoholic fatty liver disease; NASH, non-alcoholic steatohepatitis; NAS, NAFLD activity score; PCoA, principal coordinates analysis; PCR, polymerase chain reaction; SCFAs, short-chain fatty acids; TG, triglyceride; TNF-α, tumor necrosis factor–α; UPGMA, unweighted pair group method with arithmetic mean.
