# Supplementary material for: Amelioration of Non-Alcoholic Steatohepatitis by Atractylodes macrocephala Polysaccharide, Chlorogenic Acid, and Geniposide Combination Is Associated With Reducing Endotoxin Gut Leakage
Source: Front Cell Infect Microbiol. 2022 Jul 5;12:827516. doi: 10.3389/fcimb.2022.827516 (PMC9294165; doi:10.3389/fcimb.2022.827516)
Supplement: Supplementary file 1 [file DataSheet_1.doc]

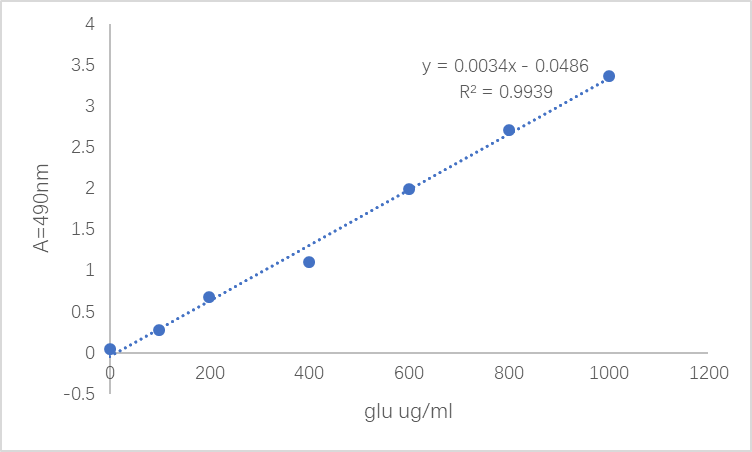


**Supplementary Figure 1. Standard curve of total carbohydrate content determined by phenol-sulfuric acid method.** A, 490 nm, the absorbance of standard samples on 490 nm, Glc μg / ml, the content of glucose.

**Supplementary Figure 2. Chemical components of *Atractylodes macrocephala* polysaccharide (**above image**) and standard (**bottom image, rhamnose, arabinose, xylose, mannose, glucose, galactose**) analyzed by gas chromatograph-mass spectrometer (GC-MS) (TRACE-DSQ, Thermo Fisher Scientific Inc., FL, USA).** Bottom image, standard analysis showed that *Atractylodes macrocephala* polysaccharide contained 5.76 % arabinose and 94.24 % glucose.


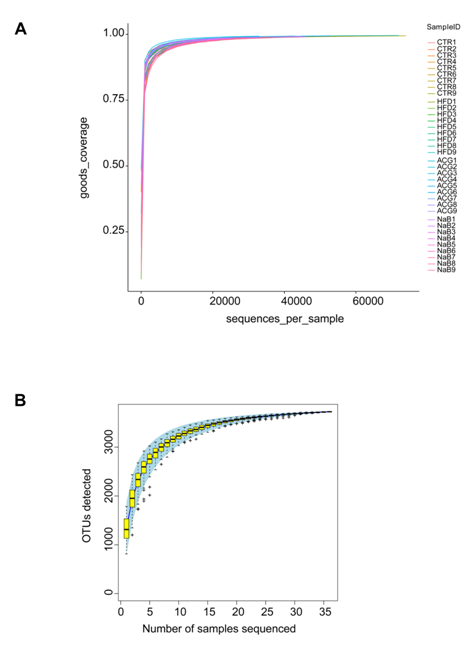


**Supplementary Figure 3 Sequencing data covers all of the species in total samples.** (A) The depth of sequencing was illustrated with Good's Nonparametric Coverage Estimator, which is more approaching to 1, the more completely the sequencing covering species in the sample. (B) Specaccum species accumulation curve indicated sequencing data covered species completely in the total samples, that the number of detected OTUs approaching to satuation as samples increasing.

**
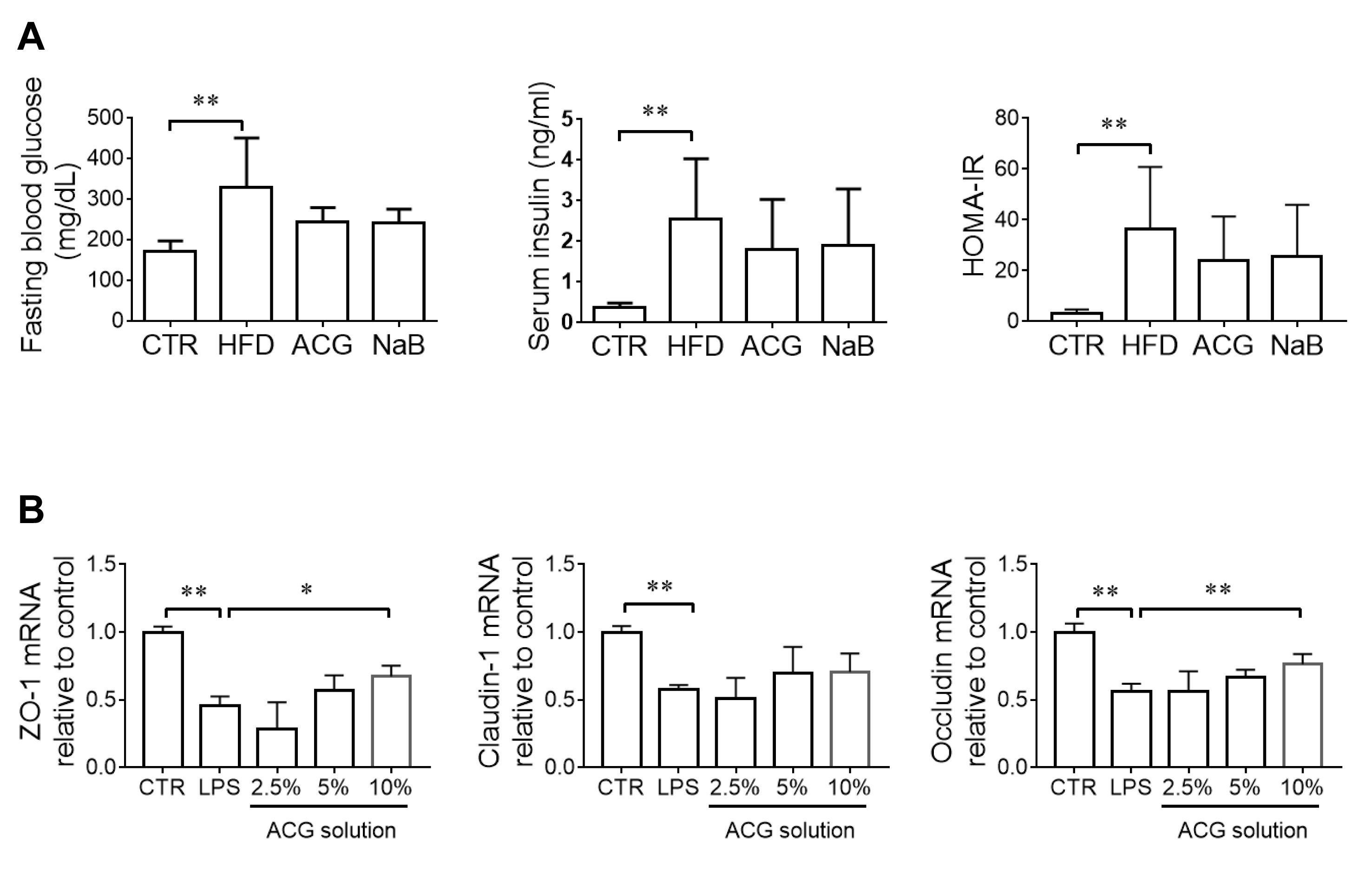
**

**Supplementary Figure 4 Insulin resistance in vivo and tight junctions in vitro.**

A, The fasting glucose and insulin levels in serum, and the homeostatic model assessment for insulin resistance (HOMA-IR). B, Tight junctions in Caco-2 cells. Caco-2 cells (Shanghai Institutes for Biological Sciences, Chinese Academy of Sciences, Shanghai, China) were cultured as described previously .

Caco-2 cells were stimulated with lipopolysaccharide (LPS) (from E. coli strain 055:B5; Sigma-Aldrich, USA) at 1 μg/ml for 24 h . Before LPS stimulation, some wells were pretreated with different concentrations (2.5%, 5%, 10%) of ACG solution for 1 h. Next, Caco-2 cells were stimulated with LPS. Untreated cells were used as the control.

In animal experiment, ACG contains *atractylodes macrocephala* polysaccharide 266.67 mg / kg body weight, chlorogenic acid 3.3 mg / kg body weight and geniposide 45 mg / kg body weight, daily. These components were dissolved in double distilled water and administrated by gavage at 1ml / 100g body weight. Therefore, ACG solution used for mice contains *atractylodes macrocephala* polysaccharide 26.667 mg / ml, chlorogenic acid 0.33 mg / ml and geniposide 4.5 mg / ml. Here, 2.5% ACG solution contains *atractylodes macrocephala* polysaccharide 0.667 mg / ml, chlorogenic acid 0.008 mg / ml and geniposide 0.1125 mg / ml. 5% ACG solution contains *atractylodes macrocephala* polysaccharide 1.33 mg / ml, chlorogenic acid 0.0165 mg / ml and geniposide 0.225 mg / ml. 10% ACG solution contains *atractylodes macrocephala* polysaccharide 2.667 mg / ml, chlorogenic acid 0.033 mg / ml and geniposide 0.45 mg / ml.

**Supplementary Table 1 The proce**dure of reagents admixture in phenol-sulfuric acid method

| Reagents | Standard 1 (ml) | Standard 2 (ml) | Standard 3 (ml) | Standard 4 (ml) | Standard 5 (ml) | Standard 6 (ml) | Standard 7 (ml) | sample (ml) |
| --- | --- | --- | --- | --- | --- | --- | --- | --- |
| 48μg / ml glucose | 0 | 0.4 | 0.7 | 1.0 | 1.3 | 1.6 | 2 | 0 |
| deionized water | 2 | 1.6 | 1.3 | 1.0 | 0.7 | 0.4 | 0 | 0 |
| CS-PS sample solution | 0 | 0 | 0 | 0 | 0 | 0 | 0 | 2 |
| 6% phenol | 1 | 1 | 1 | 1 | 1 | 1 | 1 | 1 |
| sulfuric acid | 5 | 5 | 5 | 5 | 5 | 5 | 5 | 5 |

Total carbohydrate content of *atractylodes macrocephala* polysaccharide analyzed by using the phenol-sulfuric acid method as described previously with some modification. Standard: 0-48μg / ml glucose (Sigma, MO, USA), 25 mg of *atractylodes macrocephala* polysaccharide was dissolved in 250 mL of deionized water and centrifuged at 1000 g for 10 min. The *atractylodes macrocephala* polysaccharide solution (2 mL) was added into the testing tube for detection.

Supplementary Table 2 NAFLD activity score (NAS) system

| Item | Definition | Score |
| --- | --- | --- |
| Steatosis | <5% | 0 |
|  | 5%-33% | 1 |
|  | >33%-66% | 2 |
|  | >66% | 3 |
| Lobular inflammation | No foci | 0 |
|  | < 2 foci per 200 x field | 1 |
|  | 2-4 foci per 200 x field | 2 |
|  | >4 foci per 200 x field | 3 |
| Ballooning | None | 0 |
|  | Few balloon cells | 1 |
|  | Many cells / prominent ballooning | 2 |

NAS is the unweighted sum of steatosis, lobular inflammation, and hepatocellular ballooning scores. NAS of >5 correlated with a diagnosis of NASH, and biopsies with scores of less than 3 were diagnosed as “not NASH.” ^[17]^

Supplementary Table 3 Primers used in Real-time polymerase chain reaction

| Target gene | Forward | Reverse |
| --- | --- | --- |
| Collagen I | 5’-GCTCCTCTTAGGGGCCACT-3’ | 5’-CCACGTCTCACCATTGGGG-3’ |
| Collagen IV | 5’-TCCGGGAGAGATTGGTTTCC-3’ | 5’-CTGGCCTATAAGCCCTGGT-3’ |
| CD14 | 5’-GACCTTAGTCACAATTCACTGC-3’ | 5’-GAAAGACAGATTGAGCGAGTTT-3’ |
| MyD88 | 5’-CGGAACTTTTCGATGCCTTTAT-3’ | 5’-CACACACAACTTAAGCCGATAG-3’ |
| β-actin | 5-CTCCAGAGCACCGAGAGCTA-3 | 5-ATGGGCACAGTGTGGGTGAC-3 |

Supplementary Table 4 Antibodies for western blotting and immunofluorescence staining

| Antibody | Manufacturer Catalog# | Species | Dilution | |
| --- | --- | --- | --- | --- |
|  |  |  | Western  blotting | immunofluorescence Staining |
| F4/80 | Abcam, ab6640 | rabbit | - | 1:100 |
| ZO-1 | Thermo Fisher, 40-2200 | rabbit | 1:500 | 1:100 |
| Occludin | Thermo Fisher, 40-4700 | rabbit | 1:1000 | 1:100 |
| Claudin-1 | Thermo Fisher, 71-7800 | rabbit | 1:200 | - |
| β-actin | Proteintech, 66009-1-lg | mouse | 1:5000 | - |
| Anti-rabbit IgG (DyLight^TM^ 680 Conjugate) | Cell Signaling Technology, 5366 | goat | 1:10000 | - |
| Anti-mouse IgG (H+L) (DyLight 800 4X PEG Conjugate) | Cell Signaling Technology, 5257 | goat | 1:10000 | - |
| Anti-rabbit IgG H&L (Cy3) | Abcam, ab6939 | goat | - | 1:1000 |

Supplementary Table 5 Index of alpha diversity

| Group | Observed species | Chao1 | Shannon | Simpson |
| --- | --- | --- | --- | --- |
| CTR | 951 ± 167 | 1426 ± 199 | 6.28 ± 0.61 | 0.93 ± 0.02 |
| HFD | 1038 ± 129 | 1491 ± 157 | 6.93 ± 0.38 | 0.97 ± 0.01 && |
| ACG | 781 ± 68 ** | 1134 ± 124 **& | 6.21 ± 0.26 * | 0.95 ± 0.02 |
| NaB | 1104 ± 200 | 1599 ± 237 | 6.98 ± 0.67 | 0.96 ± 0.02 & |

*p<0.05, vs HFD, **p<0.01, vs HFD; & p<0.05, vs CTR, &&p<0.01, vs CTR.

Alpha diversity of each sample was evaluated with observed species, Chao1 and Shannon index demonstrated that samples in ACG had less species diversity than that in model and normal groups. CTR, control group, HFD, high-fat diet group, ACG, the group of combination composed of *Atractylodes macrocephala* polysaccharide, chlorogenic acid and geniposide. NaB, sodium butyrate.

Reference:

[1] J. Leng, F. Huang, Y. Hai, H. Tian, W. Liu, Y. Fang, Y. Hu, and J. Peng, Amelioration of non-alcoholic steatohepatitis by Qushi Huayu decoction is associated with inhibition of the intestinal mitogen-activated protein kinase pathway. Phytomedicine : international journal of phytotherapy and phytopharmacology 66 (2020) 153135.

[2] S.K. Saha, and C.F. Brewer, Determination of the concentrations of oligosaccharides, complex type carbohydrates, and glycoproteins using the phenol-sulfuric acid method. Carbohydrate research 254 (1994) 157-67.

[3] J. Peng, X. Li, Q. Feng, L. Chen, L. Xu, and Y. Hu, Anti-fibrotic effect of Cordyceps sinensis polysaccharide: Inhibiting HSC activation, TGF-beta1/Smad signalling, MMPs and TIMPs. Experimental biology and medicine 238 (2013) 668-77.

[4] X.M. Li, J.H. Peng, Z.L. Sun, H.J. Tian, X.H. Duan, L. Liu, X. Ma, Q. Feng, P. Liu, and Y.Y. Hu, Chinese medicine CGA formula ameliorates DMN-induced liver fibrosis in rats via inhibiting MMP2/9, TIMP1/2 and the TGF-beta/Smad signaling pathways. Acta pharmacologica Sinica 37 (2016) 783-93.
